# Genome-Wide Association Studies for the Detection of Genetic Variants Associated With Daptomycin and Ceftaroline Resistance in *Staphylococcus aureus*

**DOI:** 10.3389/fmicb.2021.639660

**Published:** 2021-02-15

**Authors:** Robert E. Weber, Stephan Fuchs, Franziska Layer, Anna Sommer, Jennifer K. Bender, Andrea Thürmer, Guido Werner, Birgit Strommenger

**Affiliations:** ^1^Department of Infectious Diseases, Robert Koch-Institute, Wernigerode, Germany; ^2^Methodology and Research Infrastructure, Bioinformatics, Robert Koch-Institute, Berlin, Germany; ^3^Methodology and Research Infrastructure, Genome Sequencing, Robert Koch-Institute, Berlin, Germany

**Keywords:** GWAS, daptomycin, ceftaroline, *S. aureus*, antibiotic resistance, PLINK, SEER

## Abstract

**Background:**

As next generation sequencing (NGS) technologies have experienced a rapid development over the last decade, the investigation of the bacterial genetic architecture reveals a high potential to dissect causal loci of antibiotic resistance phenotypes. Although genome-wide association studies (GWAS) have been successfully applied for investigating the basis of resistance traits, complex resistance phenotypes have been omitted so far. For *S. aureus* this especially refers to antibiotics of last resort like daptomycin and ceftaroline. Therefore, we aimed to perform GWAS for the identification of genetic variants associated with DAP and CPT resistance in clinical *S. aureus* isolates.

**Materials/methods:**

To conduct microbial GWAS, we selected cases and controls according to their clonal background, date of isolation, and geographical origin. Association testing was performed with PLINK and SEER analysis. By using *in silico* analysis, we also searched for rare genetic variants in candidate loci that have previously been described to be involved in the development of corresponding resistance phenotypes.

**Results:**

GWAS revealed MprF P314L and L826F to be significantly associated with DAP resistance. These mutations were found to be homogenously distributed among clonal lineages suggesting convergent evolution. Additionally, rare and yet undescribed single nucleotide polymorphisms could be identified within *mprF* and putative candidate genes. Finally, we could show that each DAP resistant isolate exhibited at least one amino acid substitution within the open reading frame of *mprF*. Due to the presence of strong population stratification, no genetic variants could be associated with CPT resistance. However, the investigation of the staphylococcal cassette chromosome *mec* (SCC*mec*) revealed various *mecA* SNPs to be putatively linked with CPT resistance. Additionally, some CPT resistant isolates revealed no *mecA* mutations, supporting the hypothesis that further and still unknown resistance determinants are crucial for the development of CPT resistance in *S. aureus*.

**Conclusion:**

We hereby confirmed the potential of GWAS to identify genetic variants that are associated with antibiotic resistance traits in *S. aureus.* However, precautions need to be taken to prevent the detection of spurious associations. In addition, the implementation of different approaches is still essential to detect multiple forms of variations and mutations that occur with a low frequency.

## Introduction

*Staphylococcal aureus* is a major human pathogen that is responsible for a large number of community- and hospital associated infections worldwide (Holden et al., 2013). It causes a variety of human maladies, from minor skin and soft tissue infections to systematic and life-threatening diseases such as endocarditis, pneumonia, and septicemia (Tong et al., 2015). The introduction of penicillinase-stable β-lactam antibiotics six decades ago gave rise to the selection and spread of methicillin-resistant *S. aureus* (MRSA) (Holden et al., 2013). This resistance phenotype is mediated by horizontal acquisition of the staphylococcal cassette chromosome *mec* (SCC*mec*) and, in particular *mecA* (or its homologues *mecB/mecC*), that encodes the alternative penicillin-binding protein 2A (PBP2a) which has significantly less affinity to methicillin (Robinson and Enright, 2003).

For many years, vancomycin (VAN) administration has been considered the reference standard for the treatment of invasive MRSA infections (Micek, 2007). However, owing to an overall increase in VAN minimal inhibitory concentrations (MIC), potential adverse consequences and concern of treatment failure, the role of VAN as first-line antibiotic has become controversial in modern therapeutics (Bruniera et al., 2015). Later on, new antibiotics such as daptomycin (DAP) and ceftaroline (CPT) have been developed, showing a promising anti-MRSA activity. The cyclic lipopeptide DAP was approved in Europe by the EMA for the treatment of skin and skin structure infections (SSSI), bacteremia and right sided endocarditis in 2006/2007 (Gonzalez-Ruiz et al., 2011). The proposed mechanism of action involves the calcium-dependent integration of DAP into the bacterial cell membrane that is triggered by the binding of DAP to the negatively charged phospholipid phosphatidylglycerol (PG) (Bayer et al., 2013). Although subsequent pore formation and ion leakage have frequently been described as the cause of cell death, recent studies suggest that the interaction between DAP and fluid membrane microdomains results in cell wall defects like membrane rigidification, depolarization and the delocalization of essential membrane proteins (Muller et al., 2016; Ernst and Peschel, 2019; Gray and Wenzel, 2020). DAP resistance of *S. aureus* has frequently been associated with single nucleotide polymorphisms (SNPs) in the multi-peptide resistance factor MprF, leading to an enhanced production and translocation of the positively charged lysyl-phosphotidylglycerol (L-PG) and thereby to a repulsion of the calcium-complexed DAP (Friedman et al., 2006; Mishra et al., 2011; Peleg et al., 2012; Bayer et al., 2013; Yang et al., 2013). However, results of recent studies suggest that these mutations might instead result in an extended substrate spectrum of MprF, thereby enabling the translocation of DAP itself or membrane proteins that are critical for the activity of DAP (Ernst and Peschel, 2019).

In 2012 CPT was licensed in Europe for treating serious skin and soft tissue infections and community acquired pneumonia (AstraZeneca, 2012). The fifth-generation cephalosporin binds with high affinity to the allosteric domain of PBP2a, thereby stimulating a multi-residue conformation change that predisposes the active site of PBP2a to be inactivated by a second CPT molecule (Kosowska-Shick et al., 2010; Otero et al., 2013). The irreversible acylation of the active serine causes the protein to lose its function and finally results in loss of membrane integrity and cell death (Otero et al., 2013; Peacock and Paterson, 2015). Treatment failure is expected to be caused by mutations in either the transpeptidase or allosteric domain of PBP2a that are accompanied with decreased binding capacity of CPT or impaired protein-protein interactions between PBP2a and PBP2 (Otero et al., 2013).

Although multiple genetic determinants of CPT and DAP resistance have been described, scientists still lack a deeper understanding of the involved resistance mechanisms, because a variety of factors and processes appear to affect the antimicrobial activity of these compounds (Şlusarczyk et al., 2018; Ernst and Peschel, 2019).

Since Next Generation Sequencing (NGS) technologies have experienced a rapid development in the past 10 years, the investigation of the bacterial genetic architecture reveals high potential do dissect causal loci of antimicrobial resistance traits. With the integration of whole genome sequencing in the clinical and public health setting, gene databases and tools have been developed to allow for the assessment of genes associated with antimicrobial resistance (Feldgarden et al., 2019; Wheeler et al., 2019; Alcock et al., 2020; Bortolaia et al., 2020). However, these tools quickly fail when facing multifactorial or yet unknown resistance mechanisms. In this case, genome-wide association studies (GWAS) have become a promising option for the prediction of resistance phenotypes based on genomic data (Farhat et al., 2013, 2019; Alam et al., 2014; Chen and Shapiro, 2015). For staphylococci, GWAS have also been successfully applied to identify genetic determinants significantly associated with virulence, pathogenicity and growth plasticity (Laabei et al., 2014; Meric et al., 2018; Rong et al., 2019; Young et al., 2019). As GWAS were originally developed for human studies, microbial GWAS had to be adopted to bacterial populations, addressing confounding factors like population stratification, linkage disequilibrium, and population structure that can lead to the detection of spurious associations if not corrected properly (Falush and Bowden, 2006; Chen and Shapiro, 2015; Power et al., 2017; Farhat et al., 2019). Here we consider two strain collections of clinical *S. aureus* isolates to both identify genetic variants associated with DAP and CPT resistance and to highlight the chances and limitations of bacterial GWAS.

## Materials and Methods

### Bacterial Isolates

All isolates originated from submissions to the German National Reference Centre (NRC) for Staphylococci and Enterococci. Species confirmation was conducted by colony morphology and positive plasma coagulase reaction. Isolates have been further characterized by *spa* typing as described previously (Strommenger et al., 2008). Since DAP and CPT resistance in *S. aureus* is still rare (Roch et al., 2017; Urban and Stone, 2019), the sample sizes remained comparatively small (Power et al., 2017). A total of 48 daptomycin resistant (DAP-R) and 47 daptomycin susceptible (DAP-S) *S. aureus* isolates were selected according to Robert and Weber (2017). The DAP breakpoint for *S. aureus* according to EUCAST version 7.1^1^ was 1 mg/L (S ≤ 1 mg/L, R > 1 mg/L). The final strain collection comprised clonal lineages CC5 [clonal complex; including sequence type (ST) ST5 (*n* = 3), ST225 (*n* = 21), ST149 (*n* = 2), ST4411 (*n* = 1), and ST228 (*n* = 1)], CC8 [including ST8 (*n* = 16), ST239 (*n* = 1), and ST241 (*n* = 1)], CC15 [including ST15 (*n* = 3) and ST4410 (*n* = 1)], CC45 [including ST45 (*n* = 5), ST4409 (*n* = 1), and ST4408 (*n* = 1)], ST7 (*n* = 4), ST22 (*n* = 30), ST30 (*n* = 2), and ST398 (*n* = 2) (Supplementary Figure 1A). Both, methicillin-susceptible *S. aureus* (MSSA, *n* = 32) and methicillin-resistant *S. aureus* (MRSA, *n* = 63) were considered. Isolates were defined as MRSA on the basis of oxacillin resistance (MIC_OXA_ > 2 mg/L), cefoxitin resistance (MIC_CIX_ > 4 mg/L; CIX MICs not available for isolates collected prior to 2014) and *mecA* carriage. For CPT analysis, we selected a total of 44 CPT resistant (CPT-R) and 43 CPT susceptible (CPT-S) *S. aureus* isolates. The CPT breakpoint for *S. aureus* according to EUCAST version 7.1 was 1 mg/L (S ≤ 1 mg/L, R > 1 mg/L). The final strain collection included clonal lineages CC5 [including ST5 (*n* = 12), ST225 (*n* = 13), ST146 (*n* = 1), ST228 (*n* = 44), ST111 (*n* = 7), and ST4511 (*n* = 1)], CC8 [including ST8 (*n* = 3), ST113 (*n* = 1), ST239 (*n* = 9), ST14407 (*n* = 1), and ST1465 (*n* = 1)], ST398 (*n* = 1), ST30 (*n* = 6), and CC22 [including ST22 (*n* = 19) and ST4406 (*n* = 1)] (Supplementary Figure 1B). The Supplementary Tables 1, 2 contain detailed information for both strain collections.

### Susceptibility Testing

DAP, CPT, and OXA MICs were determined by the use of broth microdilution (BMD) according to EUCAST guidelines version 7.1 as described before (Strommenger et al., 2015; Robert and Weber, 2017). ATCC 29213 was used as quality control strain. In case of ambiguous CPT MIC results, measurements were repeated up to three times and a median was calculated. Additionally, the following clinically or epidemiologically relevant antibiotics were routinely tested: benzylpenicillin (BEN), Cefoxitin (CXI, since 2014) gentamicin (GEN), erythromycin (ERY), clindamycin (CLI), tetracycline (TET), vancomycin (VAN), teicoplanin (TEI), ciprofloxacin (CIP), trimethoprim-sulfamethoxazole (TRS), fusidic acid (FUS), rifampicin (RIF), mupirocin (MUP), phosphomycin (FOS), linezolid (LIN), moxifloxacin (MOX), and tigecycline (TIG).

### Whole Genome Sequencing and Quality Control

Mueller Hinton Bouillon (MHB) was used as growth medium for bacterial cultivation. Isolation of genomic DNA from an overnight culture of *S. aureus* was performed using the DNeasy Blood & Tissue Kit (Qiagen, Hilden, Germany) with a lysostaphin pre-treatment in 20 mM Tris/HCl, pH 8.0, 2 mM sodium EDTA, 1.2% Triton X-100 (100 μg/ml final concentration of lysostaphin). For determining purity and quantity of nucleic acids, the Eppendorf BioPhotometer (Eppendorf AG, Hamburg, Germany) and the Qubit dsDNA HS Assay Kit (Thermo Fisher Scientific, Waltham, MA, United States) were used in line with the manufacturer’s instructions. Sequencing libraries were generated with the Nextera XT DNA Library Preparation Kit (Illumina, San Diego, CA, United States) and whole genome sequencing was carried out in paired-end on a MiSeq instrument using the 2 **×** 300-cycle version 3 kit as recommended by the manufacturer (Illumina, San Diego, CA, United States). Subsequently, quality of raw sequence data was checked using FastQC version 0.11.5 (Andrews et al., 2012). To clean up raw reads we excluded poor-quality and undersized reads by applying Trimmomatic (Bolger et al., 2014) with LEADING/TAILING 3 and MINLEN 36 as default parameters. Additionally, in case of read mapping and *de novo* assembly, we used SLIDINGWINDOW 4:15 and MAXINFO 15:0.8, respectively. The quality of trimmed reads was rechecked using FastQC.

### Phylogenetic Analysis

Reference sequences were selected by the use of *refRank* version 3.0.0 (Becker et al., 2018) with a set of 142 complete *S. aureus* genome sequences available at NCBI (03/2017). *S. aureus* COL (MRSA, ST250, acc. no. NC_002951) and *S. aureus* ECT-R2 (MSSA, ST5, acc. no. NC_017343) were used as a reference strain for phylogenetic reconstruction of the DAP and CPT strain collections, respectively. As recombination events are well-known confounders of tree topology, we screened annotations of the reference genomes *S. aureus* COL and *S. aureus* ECT-R2 for mobile genetic elements and genes associated with drug resistance which were then removed (cut out) from the reference genomes (Supplementary Table 3). In the following, we will refer to these sequences as modified reference sequences. Subsequently, trimmed paired-end reads were read-aligned to the corresponding modified reference genomes using our in-house pipeline *batchMap* as described previously (Halbedel et al., 2018). Based on the generated multiple consensus alignment SNPs were filtered using *SNPFilter* version 3.0.0 (Becker et al., 2018) with an exclusion distance of 150 bp. By setting an exclusion distance SNPs within spatial proximity (150 bp) to each other were excluded to additionally prevent recombination events to alter the phylogeny. Resulting SNP-alignments were used with Geneious version 8.1.7 (Biomatters Ltd., Auckland, New Zealand) to calculate neighbor joining consensus trees with bootstrap support (100 replicates). For illustration purpose iTOL v3.2.4 was used (Letunic and Bork, 2016).

### Genome-Wide Association Studies (GWAS)

#### Genome Reconstruction and SNP Calling

For the identification of SNPs, trimmed Illumina reads of isolates belonging to the DAP strain collection were aligned to *S. aureus* COL by the use of *batchMap*. Since *S. aureus* ECT-R2 lacks SCC*mec*, *S. aureus* 04-02981 [acc. no. NC_017340, MRSA, ST225, SCC*mec* type II (2A)] was used as a reference sequence for CPT PLINK analysis. Alignments of consensus sequences were reduced to variant positions using *SNPFilter* without applying an exclusion distance. As SEER is an alignment-free method, high paired-end reads were *de novo* assembled using A5-miseq 20150522 with default parameters (Coil et al., 2015).

#### PLINK Analysis

Multi-sequence alignments were used to create input files for PLINK v1.90b3.31^2^ (Purcell et al., 2007). Since PLINK is unable to utilize triallelic SNPs, we replaced the minor with the major variant. Prior to PLINK analysis, we screened for isolates with more than 10% missing genotypes and excluded SNPs on the basis of minor allele frequency (MAF < 5%) or missing genotype data (call rate < 90%). Furthermore, a Z score was calculated to detect population outliers, which were subsequently excluded from further analysis (standard deviation > 3; Supplementary Table 4). To account for population stratification, subpopulations within the overall population were identified with hierBAPS v6.0 (Cheng et al., 2013) and covariates were used with the Cochran-Mantel-Haenszel test (CMH) for 2 × 2 × K stratified tables. In order to search for the evidence of systematic bias a quantile-quantile (Q-Q) plot was constructed that compares the observed and expected *p-*value (-log_10_ transformed) under the null hypothesis of no true association. In case of substantial deviation of the observed *p*-values from the null hypothesis, we used genomic control (GC) to control for the confounding effects of population stratification. Finally, we created a Manhattan Plot illustrating the genome wide significance levels of corresponding SNPs in relation to their genome position. For illustration purposes we excluded SNPs with a -log_10_ *p-*value of 0.

#### Sequence Element Enrichment Analysis (SEER)

SEER identifies k-mers that are significantly enriched in a phenotype of interest and includes an alignment-free correction to account for population structure (Lees et al., 2016). For association analysis, we used SEER v.1.4.1. K-mers were counted from *de novo* assembled contigs by the use of the single-core implementation of frequency-based substring mining (fsm-lite; options -s 5 and -S 95)^3^. To create a matrix representing population structure, kmds (control for population structure) was performed with the *no filtering* option and a total of 200.000 k-mers. The matrix was then used to run SEER association statistics. Subsequently, we excluded k-mers with a MAF < 5%, a negative beta (ß_1_ ≤ 0) and a length < 20 bp. Post-association filtered k-mers were used for the construction of Q-Q plots. In general, we used the likelihood ratio test (lrt) *p*-value for further downstream analysis. In case of strong population stratification, we preferred the Wald-test over the LRT. Those reaching the Bonferroni corrected significance thresholds were finally mapped to annotated reference genomes (see above).

#### Searching for Low Frequency and Rare SNPs

To detect rare mutations (MAF < 5%) in candidate genes, we used ancestral state reconstruction in Mesquite v2.75^4^ (Maddison and Maddison, 2017).

#### SCC*mec* Analysis

Elements of the staphylococcal cassette chromosome (SCC*mec*) were identified using SCC*mec*Finder v1.0^5^ (Kaya et al., 2018). The web-based tool uses *de novo* assembled reads (contigs) and defines the distinct position of each SCC*mec* associated gene. Therefore, we were able to extract corresponding regions for each isolate which we subsequently aligned with progressive Mauve algorithm (Darling et al., 2004).

## Results and Discussion

### Identification of DAP-R Associated Mutations in *S. aureus*

#### Phylogenetic Reconstruction and Prevention of Population Stratification

For the identification of DAP-R associated mutations, we established a strain collection containing 95 *S. aureus* isolates, of which 48 were resistant to DAP (Supplementary Table 1). The strain collection comprised 4 CCs and 17 STs that predominantly mirrored the distribution of CCs and STs in Europe and, in particular, in Germany (Grundmann et al., 2010; Asadollahi et al., 2018; Supplementary Figure 1A). Within most CCs and STs we observed a well-balanced distribution of resistance phenotypes, that could be achieved by selecting and matching isolates, whenever possible, according to their clonal background (ST/CC), isolation date and geographical origin (Supplementary Figures 1A, 2). In microbial GWAS, a homogeneous distribution of cases and controls reduces the risk of detecting spurious associations (associations that are due to relatedness rather than a true association with the phenotype of interest). These types of associations usually occur as a consequence of population stratification (PS) which refers to a situation in which members of a group of interest are on average more closely related to each other, than to the rest of the wider population (Chen and Shapiro, 2015).

To address systematic bias caused by population stratification in PLINK analysis, subpopulations within the overall population were inferred with hierBAPS (Cheng et al., 2013). Altogether, five hierBAPS sub-clusters could be identified that mirrored the phylogenetic relatedness of 95 *S. aureus* isolates (Supplementary Figure 2). These sub-clusters were then used as covariates with the CMH and a total of 66.667 SNPs (relative to the reference genome *S. aureus* COL), allowing to test for associations conditional on the population structure (Chen and Shapiro, 2015). This approach has already shown to reliably reflect the phylogenetic relatedness of bacterial populations and to efficiently control for population stratification in microbial GWAS (Chewapreecha et al., 2014; Howell et al., 2014; Chen and Shapiro, 2015; Weinert et al., 2015; Mobegi et al., 2017). To test whether systematic inflation occurred due to population stratification, we constructed a Q-Q plot and calculated the genomic inflation factor lambda (λ_GC_). In a Q-Q plot, most *p-*values should follow a uniform distribution, with few SNPs producing significant *p-*values at the end of the line (Power et al., 2017). Furthermore, the λ_GC_ should be around 1 as values higher than 1.05 are seen as genome-wide inflation (Price et al., 2010). The constructed Q-Q plot of 14.525 adjusted PLINK variants revealed that most of the *p-*values fit the expected distribution (λ_GC_ = 1), with few SNPs deviating at the end of the tail (Supplementary Figure 4A).

In contrast to PLINK, SEER controls for clonal population structure by distance matrix construction and subsequent multidimensional scaling (Lees et al., 2016). This method is analogs to the principal component analysis (PCA) used in human association studies, but with the advantage of being directly applicable to k-mer counting instead of relying on core gene alignment or SNP calling (Price et al., 2010; Lees et al., 2016). With the ability to capture various types of genetic variations, SEER has successfully been used to identify genetic markers in *Burkholderia pseudomallei* (Chewapreecha et al., 2017) and to investigate host adaption of *Streptococcus suis* (Weinert et al., 2019). By applying fsm-lite we counted 10 M k-mers that were then tested for association. As recommended by Lees^6^, we used the likelihood ratio test (lrt) *p-*value for further downstream analysis. The Q-Q plot of the resultant *p-*value distribution of 3 M quality-filtered k-mers demonstrated no severe deviation of the observed from the expected *p-*values (Supplementary Figure 4C), indicating that correction for clonal population structure was appropriate.

#### PLINK GWAS for the Identification of SNPs Associated With DAP Resistance

**FIGURE 1:**
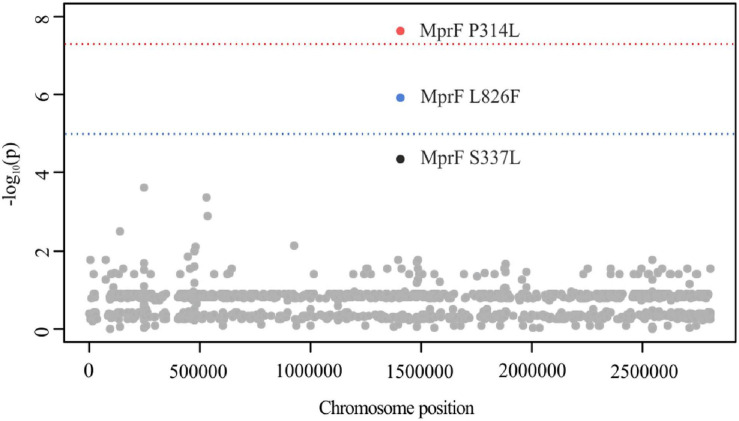
Manhattan plot illustrating DAP PLINK results. The -log_10_ unadjusted *p*-values of identified SNPs are plotted against the entire length of the chromosome of *S. aureus* COL. The blue line indicates suggestive association (*p* = 1 × 10^–5^), while the red line indicates the genome-wide significance threshold (*p* = 5 × 10^–8^). For illustration purposes, SNPs with a -log_10_ *p*-value of 0 have been excluded (*n* = 14.527).

Out of 14.525 SNPs, PLINK identified MprF P314L (*mprF* locus tag: SACOL_RS07105, *p* = 2.39 **×** 10^–8^) and L826F (*p* = 1.25 **×** 10^–6^) to be associated with DAP resistance (Figure 1). Although not reaching the suggestive association threshold, we additionally detected MprF S337L (*p* = 4.61 × 10^–5^) (Figure 1). Both P314L and S337L are located in the central bifunctional domain of MprF that is involved in L-PG synthesis and flipping, while MprF L826F is found in the C-terminal catalytic synthase domain of the protein. In previous studies, these amino acid substitutions (AAS) were suspected to be linked with a gain-of-function phenotype, either in terms of increased synthesis or enhanced translocation of L-PG, resulting in the electrostatic repulsion of calcium-complexed DAP (Friedman et al., 2006; Bayer et al., 2013, 2015; Steed et al., 2013; Heidary et al., 2017). These findings were, however, not consistent and other seemingly MprF-independent mechanisms, such as the increase in D-alanylation of teichoic acid, have been described in clinical DAP-R isolates (Bertsche et al., 2011; Mishra et al., 2014). By engineering *mprF* substitution mutants in *S. aureus*, Ernst et al. (2018) showed that the *mprF* signature mutations S295L, P314L, S337L, I420N, and L826F had no impact on cell surface charge and were not sufficient to confer DAP resistance on their own. Therefore one may suggest that additive genetic variations contribute to DAP resistance that might have evolved prior to or acquired during antibiotic treatment as could be shown in early passaging experiments by Friedman et al. (2006). Interestingly, a recent study suggests that the *mprF* signature mutations might lead to an extended substrate spectrum of MprF by weakening the intramolecular interaction between the flippase and the synthase domain of the protein (Ernst and Peschel, 2019). These structural changes may enable the translocation of DAP or critical membrane-embedded molecules from fluid microdomains, thereby conferring DAP resistance. In a clinical context, although rarely reported, few case reports describe the *in vivo* acquisition of daptomycin resistance in *S. aureus* during daptomycin therapy with subsequent clinical failure for bacteremia/endocarditis that could be associated with mutations in *mprF* (including T345A and L826F) (Murthy et al., 2008; Sotillo et al., 2016). In addition, a study conducted by Richards et al. (2015) suggests *mprF* mutations to be involved in *S. aureus* persistence during complex bacteremia by enhancing bacterial fitness and immune evasion.

#### SEER GWAS for the Identification of k-mers Associated With DAP Resistance

Due to the high degree of genome plasticity in bacterial populations as well as the need for reference sequences, PLINK is limited in the detection of causal genetic variations that are associated with differences in gene content, recombination events or variable promotor architectures (Lees et al., 2016). To overcome typical shortcomings of SNP based association tools, we additionally used SEER as an alignment free tool. This procedure is also recommended by San et al. (2019) who suggest testing for multiple forms of variations, especially when the type of variation responsible for the phenotype of interest is not known *a priori*.

**FIGURE 2:**
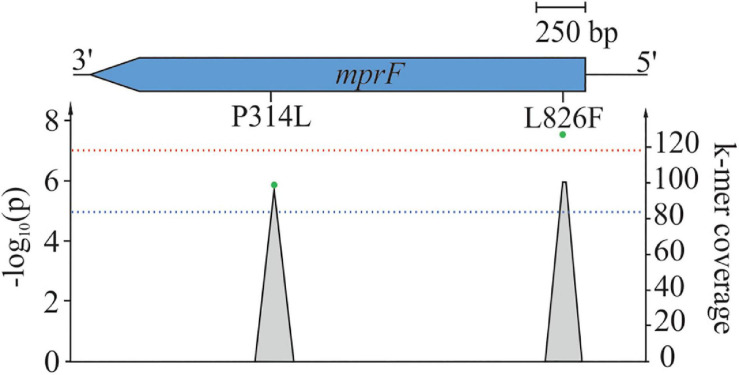
Mapping of significant k-mers against *S. aureus* COL. One **hundred ninety-eight** significant k-mers were mapped against the reference *S. aureus* COL. Gray-shaded elements illustrate k-mer coverage at corresponding loci while green dots represent adjusted (lrt) *p*-values of mapped k-mers. The blue line indicates suggestive association (*p* = 1 × 10^–5^), while the red line indicates the genome-wide significance threshold (*p* = 5 × 10^–8^).

Using SEER, we identified 198 significant k-mers that were mapped to the reference sequence of *S. aureus* COL. All k-mers were found to be located within *mprF* exhibiting either AAS P314L (*p* = 1.2 × 10^–6^) or L826F (*p* = 2.4 × 10^–8^) (Figure 2; Ng and Henikoff, 2003). The AAS P314L did not reach genome wide significance (Figure 2). This is most likely due to lineage specific mutations within *mprF* which prevent “identical” k-mers from accumulating at specific regions. Isolates from CC45 (carrying MprF P314L) have a significantly different *mprF* nucleotide sequence when compared to isolates of the wider population (Supplementary Figure 7). Therefore, k-mers harboring P314L were split into separate clusters, thus leading to a shift in significance levels. Additionally, we examined SEER results for additional MprF AAS and found S337L with k-mer *p-*values close to the suggestive association threshold (*p* = 2.4 × 10^–5^).

#### Distribution of MprF AAS

**FIGURE 3:**
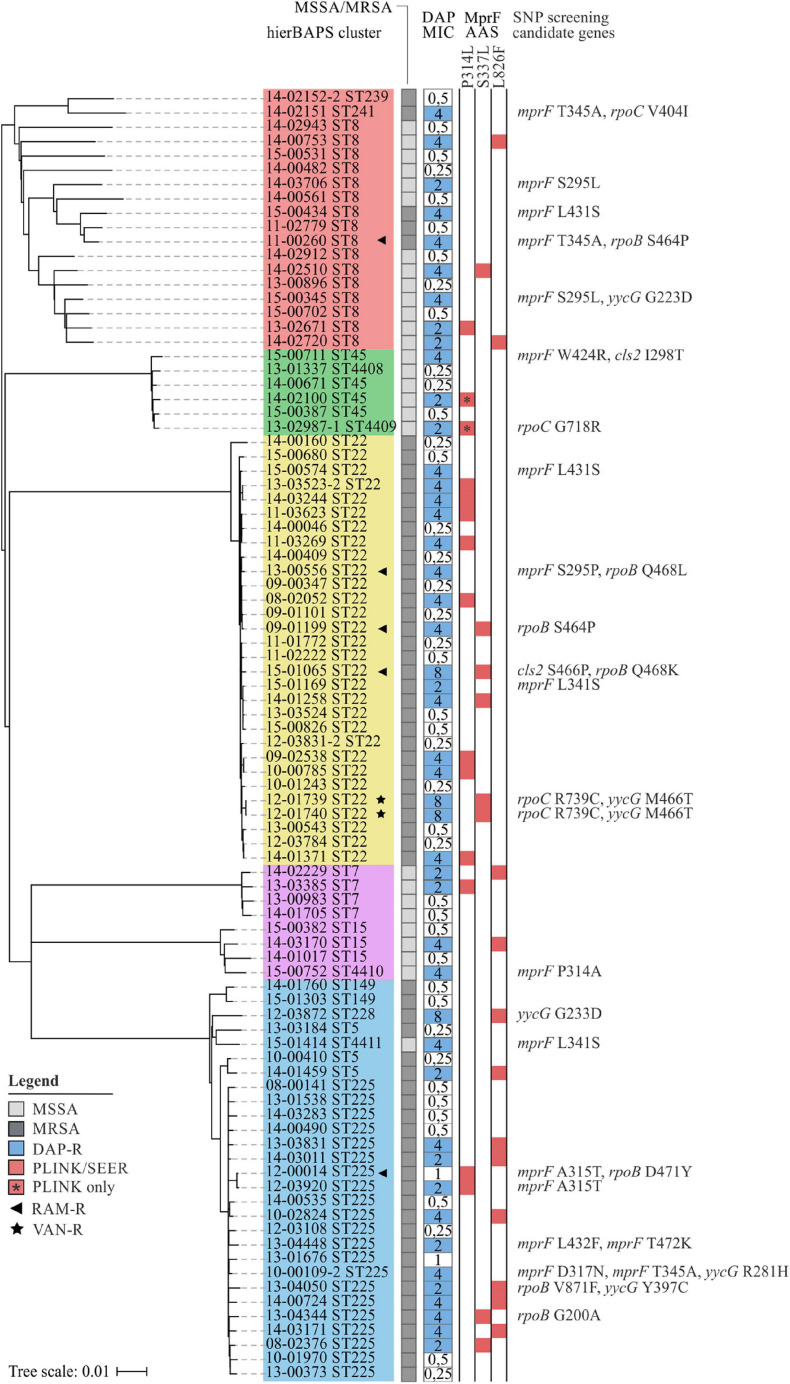
Phylogenetic relation and genotypes of DAP-R *S. aureus* included in this study. On the left, a neighbor-joining tree based on 7.342 SNPs represents the phylogenetic relatedness of 90 *S. aureus* isolates. Identified hierBAPS subclusters are color-coded. Dark and light shaded grayish boxes represent MRSA and MSSA isolates, respectively. Blue boxes point to DAP resistance. Corresponding numbers refer to DAP MICs as determined by BMD. Red-shaded squares indicate the presence of AAS that could be identified by SEER and/or PLINK analysis. On the right, AAS in candidate genes are shown as identified by the use Mesquite analysis.

As can be seen from Figure 3, MprF P314L, S337L, and L826F were predominantly restricted to DAP-R isolates and showed a fairly homogeneous distribution among clonal clusters (Figure 3). The independent emergence of identical mutations on multiple ancestral branches points to a convergent evolution, which is a strong indicator for positive selection, e.g., by antibiotic pressure (Shapiro et al., 2009). Despite the even distribution, we observed a slight tendency toward an association of MprF AAS with certain clonal lineages (e.g., P314L with isolates of CC22 or L826F with isolates of CC5) (Figure 3). This phenomenon has already been described in a previous study in which the predominance of genetic modifications associated with the vancomycin-intermediate *S. aureus* (VISA) phenotype could be linked with specific genetic backgrounds (McGuinness et al., 2017). Thus, although associated mutations may explain the phenotype of interest in the studied population, the results cannot necessarily be applied to the wider population.

#### Searching for Rare Variants Associated With DAP Resistance

For some isolates no *mprF* mutations could be identified by GWAS (*n* = 13; Figure 3). Therefore, we used the software package Mesquite (Maddison and Maddison, 2017) to manually search for rare genetic variants in *mprF*, *cls2* (SACOL_RS10875), *rpoB* (SACOL_RS03050), *rpoC* (SACOL_RS03055), *yycF* (SACOL_RS00120), *yycG* (SACOL_RS00125), *pgsA* (SACOL_RS06645), and *ddl* (SACOL_RS10850), as these loci have previously been described to be associated with DAP resistance (Friedman et al., 2006; Peleg et al., 2012; Bayer et al., 2015; Berti et al., 2015).

At least one MprF related AAS could be identified in each DAP-R isolate (Figure 3), three of which, A315T, D317N, and L432F, have not been described until now. This observation emphasizes the importance of MprF in the development of DAP resistance in *S. aureus*. Additionally, our results suggest that *mprF* mutations do not accumulate (Figure 3) but rather occur as individual hot-spot mutations. These findings are supported by Yang et al. (2018) who showed that an accumulation of point mutations paradoxically caused a reduction in DAP MICs, positive surface charge and L-PG synthesis.

For Cls2 we detected the AAS I298T (15-00711) and S466P (15-01065) that are located in close proximity to the putative cadiolipin synthase domains at residues 229–256 and 407–434 (Figure 3; Peleg et al., 2012). AAS in Cls2 and varying levels of *cls2* transcription have already been suggested to be involved in the development of DAP resistance in *S. aureus*, either alone or in combination with *mprF* mutations (Camargo et al., 2008; Peleg et al., 2012; Jiang et al., 2019; Lasek-Nesselquist et al., 2019). Interestingly, within our strain collection we found a DAP-R isolate (MIC = 8 mg/L; median of all DAP-R isolates = 4 mg/L) harboring MprF S337L, Cls2 S466P, and RpoB Q468K (15-01065; Figure 3), indicating that a combination of mutations in different loci might lead to synergistic effects. The role of *cls2* in establishing DAP resistance was also supported by studies of clinical *Enterococcus* isolates in which *cls2* mutations could be associated with elevated DAP MICs (Arias et al., 2011; Palmer et al., 2011). These changes most likely result in an altered protein function and shifted PG:cardiolipin ratios in the bacterial cell membrane, thereby leading to impaired DAP penetration and membrane disruption (Miller et al., 2016).

As could be seen for the ST22 isolate 15-01065, *rpoB* mutations might be involved in the development of DAP resistance (Figure 3). In a study conducted by Friedman et al. (2006) multiple genetic changes in laboratory-derived *S. aureus* could be identified that correlated with increased DAP MICs. After serial passaging, the authors observed not only mutations in *mprF, yycG* and *rpoC* but also *rpoB.* Furthermore, Cui et al. (2006) described a *rpoB*-mediated resistance conversion that was accompanied by an increase in DAP and vancomycin (VAN) MICs. In our study, we detected the RpoB AAS G200A (13-04344), S464P (11-00260, 09-01199), Q468K (15-01065), Q468L (13-00556), D471Y (12-00014), and V871F (13-04050) (Figure 3). While G200A and V871F have not been described until now S464P, Q468K/L, and D471Y are known to be located within the cluster 1 of the rifampicin resistance mutation site and could thus be associated with rifampicin resistance (Figure 3 and Supplementary Table 1). A correlation of the latter mutations with DAP resistance is therefore unlikely (Wichelhaus et al., 1999).

Within our strain collection, we observed two DAP-R isolates that additionally were VAN resistant (VAN MIC = 4 mg/L) (Figure 3). Increased DAP MICs are often seen in VISA isolates that are commonly characterized by thickened cell walls (Cui et al., 2006; Julian et al., 2007). Because DAP is large in molecular size, thickened peptidoglycan layers may hinder the lipopeptide from reaching its antimicrobial target and might therefore facilitate the development of a dual resistance to VAN and DAP (Cui et al., 2006). In addition to mutations in *mprF*, both isolates showed genetic variations within *rpoC* (R739C) and *yycG* (M466T) (Figure 3). The two-component regulator YycFG (also known as WalKR) is known to be involved in the positive regulation of genes associated with cell wall metabolism. Several studies have shown that mutations within *yycFG* contribute to thickening of cell walls, which are thought to result from reduced expression of the major autolysins AtlA and LytM (Friedman et al., 2006; Howden et al., 2011; Patel et al., 2011; Shoji et al., 2011; Hafer et al., 2012). As could be seen for *rpoB* mutations, genetic variations in *rpoC* may indirectly influence the expression of genes involved in cell wall biosynthesis, thereby leading to altered cell surface charges and cell wall thickening (Cui et al., 2006). A recently published study described increased cell wall thickness for a clinical *S. aureus* isolate with cross-reduced susceptibility to DAP and VAN that was likely to be associated with *mprF* W424R (Thitiananpakorn et al., 2020). Although we detected this AAS in one DAP-R isolate (Figure 3), no increased VAN MICs could be observed (VAN MIC ≤ 1 mg/L). Since cell wall thickness has not been described as a common feature of clinical DAP-R isolates, Thitiananpakorn et al. (2020) further postulated that *mprF* mediated alterations in surface charges directly affect susceptibility to both DAP and VAN. These results are somewhat contradictory to our observations, as elevated VAN MICs were observed in only two DAP-R isolates (Figure 3). Consequently, it remains to be investigated to what extent *mprF* mutations—alone or in combination with *yycFG* mutations—are effectively related to this cross-resistance phenotype. Of note, as no data on therapy was available, we are unable to judge whether these mutations were due to previous VAN or DAP therapy in the affected patients.

Genes *ddl*, *pgsA*, and *yycF* exhibited no mutations previously associated with DAP resistance.

Interestingly, MSSA and MRSA isolates showed identical MprF AAS with no characteristic patterns detectable (Figure 3). In contrast to MRSA-related infections, physicians have numerous treatment options available for MSSA-related infections (David and Daum, 2017). Thus, MSSA isolates were unlikely to be exposed to DAP therapeutically. Consequently, MSSA might have undergone selection pressures that trigger the same mechanisms of resistance as DAP. Previous studies suggest, that both cationic host defense peptides (CHDPs) and DAP trigger the same mechanisms of resistance and that the exposure of *S. aureus* to CHDPs is likely to facilitate the development of DAP resistance (Mishra et al., 2011, 2012). In addition, Renzoni et al. (2017) showed that exposure of *S. aureus* to the antiseptic polyhexanide resulted in the selection of mutants possessing *mprF* mutations and thus in cross-resistance between the antiseptic agent and clinically used antibiotics.

### Identification of CPT-R Associated Mutations in *S. aureus*

#### Phylogenetic Reconstruction and Detection of Population Stratification

The established strain collection comprised a total of 44 CPT-R and 43 CPT-S isolates (Supplementary Table 2) that belonged to 3 CCs and 15 STs (Supplementary Figure 1B). Similar to the DAP strain collection, the involved CCs and STs predominantly mirrored the distribution of clonal lineages in Europe and, more precisely, in Germany. As indicated by phylogenetic analysis, we observed the formation of distinct subclusters that lacked sufficient amounts of susceptible counterparts (Supplementary Figure 3). This is due to the fact, that isolates frequently had to be matched on the basis on CCs when no STs could be derived from *spa-*typing. Thus, although we observed a homogenous distribution of CPT resistance phenotypes within the overall CC5 clade (Supplementary Figure 1), the clustering of CPT-R isolates belonging to ST228 (CC5) and ST111 (CC5) was obscured by our sampling strategy (Supplementary Figure 1). Therefore, future studies should focus on adopting sampling strategies as described by Farhat et al., in order to both minimizing the impact of population structure and increasing the power of association studies (Farhat et al., 2014).

In order to identify SNPs associated with CPT resistance, a total of 45.989 SNPs (relative to *S. aureus* 04-02981) were analyzed with the CMH and five hierBAPS sub-clusters (Supplementary Figure 3). Although few studies described the CMH to occasionally overcorrect for population structure, we observed genome-wide inflation in the Q-Q plot of 6.543 adjusted PLINK variants (λ_GC_ = 2.11) (Supplementary Figure 5A) (Chen and Shapiro, 2015; Lees et al., 2016). Therefore, we used GC adjusted *p-*values for further downstream analysis (Supplementary Figure 4B). This method normalizes all *p-*values by the single inflation factor λ, which is the observed median chi-square divided by the expected median chi-square with 1 degree of freedom (Chen and Shapiro, 2015). The high inflation is most likely due to the close relationship of CPT-R isolates within the ST228 and ST111 clusters (Supplementary Figure 3). Previous studies have already shown that these lineages are usually associated with elevated CPT MICs and that CPT resistance in ST228 isolates had already been observed prior to the introduction of CPT in the clinical setting (Kelley et al., 2015; Strommenger et al., 2015). Therefore, the implementation of successful clonal lineages poses a unique challenge for microbial GWAS, as these phenotypic lineage-level differences need to be accounted for Lees (2017) and Power et al. (2017). This is even more relevant when investigating highly clonal pathogens such as *Mycobacterium tuberculosis*, where the entire genome is in strong linkage, further preventing the fine-mapping of causal loci (Chen and Shapiro, 2015).

Using SEER, we counted 10 M k-mers of which 4.2 M remained after association testing and post-association filtering. As we detected an early separation of the observed from the expected lrt *p-*values in the constructed Q-Q plot (Supplementary Figure 5B), we favored the Wald- of lrt-correction. Although most of the Wald-corrected *p-*values follow a uniform distribution, GWAS power was reduced significantly (Supplementary Figure 4D).

#### Population Stratification Prevents GWAS From Identifying Causal Variants

**FIGURE 4:**
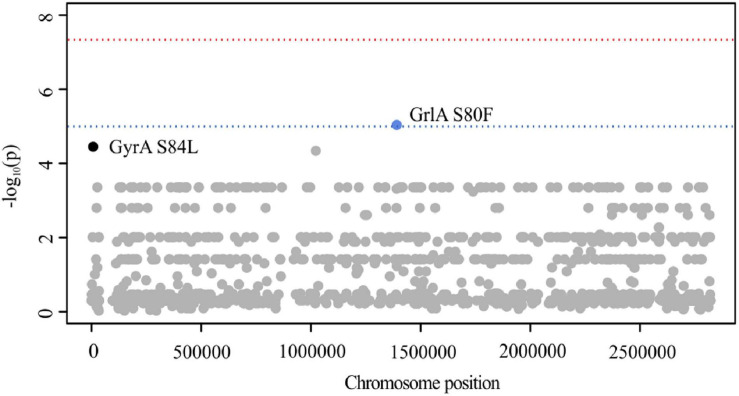
Manhattan plot illustrating CPT PLINK results. The -log_10_ GC-corrected *p-*values of identified SNPs are plotted against the chromosome position of *S. aureus* 04-02981. The blue line indicates suggestive association (*p* = 1 × 10^–5^), while the red line indicates genome-wide significance threshold (*p* = 5 × 10^–8^).

Out of 6.543 SNPs, PLINK analysis identified GrlA S80F (GC adjusted *p* = 9.05 × 10^–8^, locus tag: SA2981_RS06930) to be suggestively associated with CPT resistance (Figure 4). Close to the genome-wide significance threshold, we additionally observed GyrA S84L (GC adjusted *p* = 3.71 × 10^–7^, locus tag: SA2981_RS00030) (Figure 4). As these mutations are known to mediate ciprofloxacin (CIP) resistance in *S. aureus* (Jones et al., 2000; Kwak et al., 2013), we screened all isolates for the corresponding resistance phenotype. Indeed, we identified 95% (*n* = 42) of CPT-R and 40% (*n* = 16) of CPT-S isolates to be CIP-R, carrying either S80F or S84L (Supplementary Table 5). These observations are in line with results of previous studies, showing that dominant hospital-associated MRSA lineages are almost universally resistant to CIP and that this resistance phenotype contributes to the selection and survival of *S. aureus* (Knight et al., 2012; Layer et al., 2019). These results stress the importance of carefully verifying putative variants in order to avoid the detection of false positive correlations caused by genetically linked features as can be frequently seen in hospital-associated pathogens like *S. aureus*, *E. coli*, *E. faecium*, *M. tuberculosis*, and *A. baumanii* (Struelens, 1998; Cornejo-Juarez et al., 2015; Tacconelli et al., 2018). Using SEER analysis, 10 M k-mers were tested for CPT-R association. However, due to the stringent correction for population stratification, no significant k-mers remained (*p* < 1 × 10^–5^).

#### Searching for Rare Variants in Essential SCC*mec* Elements

**FIGURE 5:**
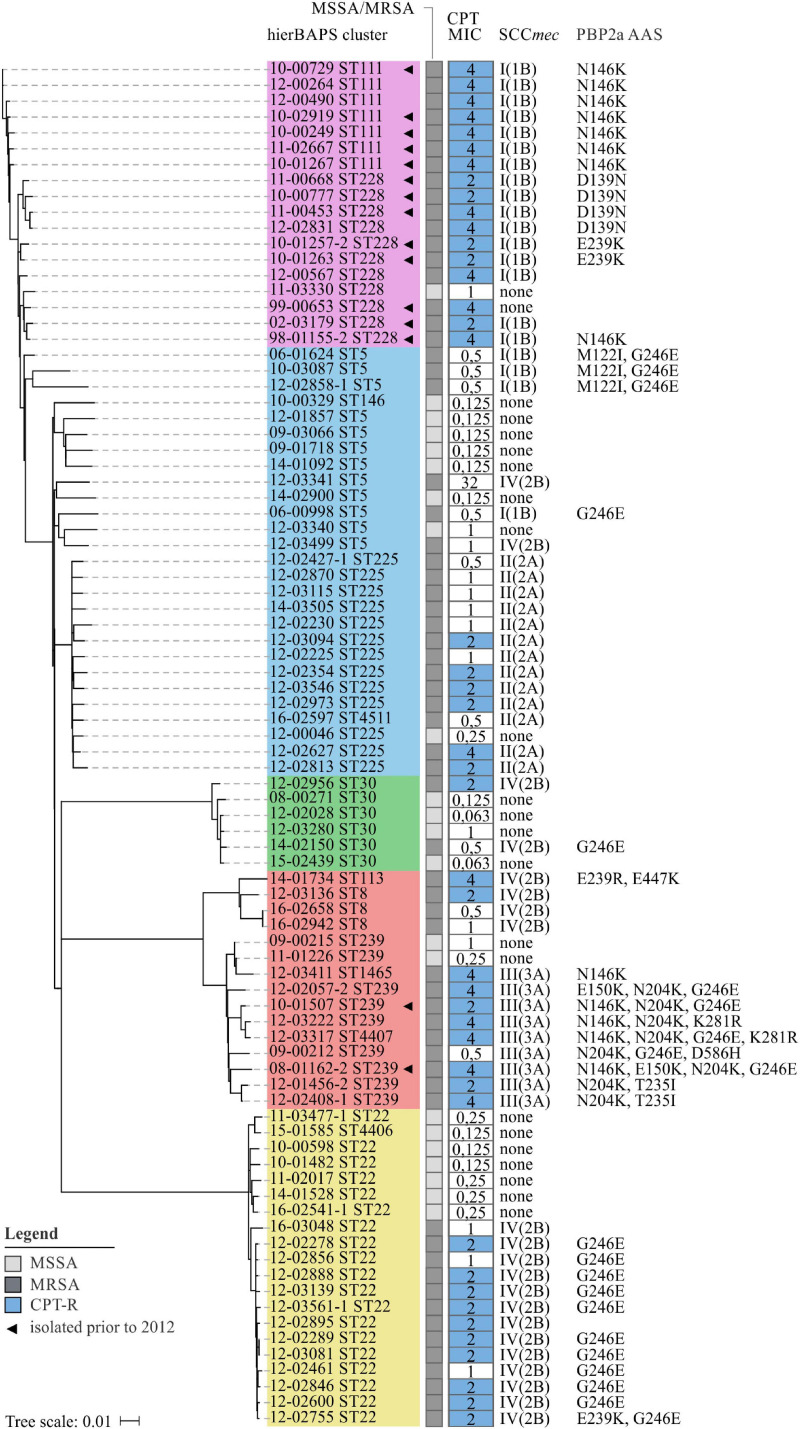
Phylogenetic relation and genotypes of CPT-R *S. aureus* included in this study. On the left, a neighbor-joining tree based on 4.344 SNPs represents the phylogenetic relatedness of 86 *S. aureus* isolates. Identified hierBAPS subclusters are color-coded. Dark and light shaded grayish boxes represent MRSA and MSSA isolates, respectively. Blue boxes point to CPT resistance. Corresponding numbers refer to CPT MICs as determined by BMD. On the right, AAS in PBP2a are shown as identified by the use of manual *in silico* analysis.

To further search for genetic variants that are putatively associated with elevated CPT MICs, the nucleotide sequences of essential SCC*mec* elements (*mecA, mecR1, ΔmecRI*, and *mecI*) were extracted from *de novo* assembled contigs. Detailed sequence analysis revealed multiple SNPs within *mecA* (D139N, N146K, E150K, N204K, T235I, E239K/R, G246E, and K281R; Figure 5), but only few were found within the regulatory genes *mecR1*, *ΔmecRI*, and *mecI* (Supplementary Table 6). The majority of AAS observed in *mecA* were restricted to the non-penicillin-binding (nPBD) domain of PBP2a (AAS 27–326) (Lim and Strynadka, 2002). These observations are in line with investigations of Lahiri et al. (2015) who found CPT-R associated substitutions to be predominantly located in or close to a structural groove of the nPBD (including D139N, N146K, E150K, N204K, T235I, and E239K). Already back in 2013, Otero et al. (2013) have shown that CPT can bind to this allosteric domain, resulting in a multiresidue conformational change of PBP2a, the opening of the active site and the irreversible acylation of the active serine by a second CPT molecule. Consequently, it has been presumed that mutations within the allosteric domain of PBP2a can lead to a disruption of salt bridge interactions that are crucial for the allosteric response, leaving the active site occluded (Otero et al., 2013). Furthermore, Alm et al. (2014) postulated that mutations in the nPBD of PBP2a might lead to a destabilization of protein-protein interactions between PBP2a and PBP2, presumably promoting the interaction with alternative PBPs, like PBP4, which has been shown to have a low affinity for CPT (Moisan et al., 2010). The *mecA* AAS G246E could not only be detected within CPT-R (*n* = 13) but also CPT-S (*n* = 7) isolates (Figure 5). Consequently, we hypothesize that this AAS on its own is unlikely to be involved in the development of CPT resistance in *S. aureus.* This assumption is supported by previous studies, showing that *S. aureus* with *mecA* G246E did not exhibit increased CPT MICs (Alm et al., 2014; Lahiri et al., 2015; Schaumburg et al., 2016). The G246E AAS in particular demonstrates that identical *mecA* mutations are often associated not only with one but several SCC*mec* types (Figure 5), suggesting that these mutations emerged after the acquisition of SCC*mec* rather than being linked with the dissemination of specific SCC*mec* types. The only change that is located near the cephalosporin-binding pocket of the penicillin-binding domain of PBP2a is the AAS E447K (Figure 5). This substitution is presumed to directly influence the binding of CPT by forming a new salt bridge with the neighboring Gluc_460_ residue (Alm et al., 2014). The diversity of *mecA* mutations clearly illustrates why SEER was limited in the detection of significant associations. In this case, k-mers were split into clusters of lower frequency, which drastically reduced significance levels and prevented the detection of corresponding mutations.

For some isolates we neither detected mutations within *mecA* nor within the regulatory elements of SCC*mec* (Figure 5). Therefore, we performed *in silico* analysis to search for mutations in *pbp1* (SACOL_RS06115), *pbp2* (SACOL_RS07590), *pbp3* (SACOL_RS08205), *pbp4* (SACOL_RS03595), *gdpP* (SACOL_RS00090), *arcB* (SACOL_RS13910), *pp2C* (SACOL_RS10765), *clpX* (SACOL_RS08790), and *rho* (SACOL_RS11055), as these loci have either been described to bind ceftaroline or to play a functional role in the development of β-lactam resistance in *S. aureus* (Banerjee et al., 2010; Moisan et al., 2010; Chan et al., 2015; Greninger et al., 2016). However, with the exception of PBP1 P360S and PBP2 A382T (12-02627; ST225), no putative resistance-associated AAS could be detected. Thus, still unknown genetic variants seem to influence the development of CPT resistance in *S. aureus* which remain to be identified.

We detected 15 CPT-R *S. aureus* isolates that were collected prior to the licensing of CPT in Europe which mainly belonged to clonal lineages ST111 and ST228 (Figure 5). Of these, eight isolates have already been described in a study by Strommenger et al. (2015). Also Kelley et al. (2015) reporteded CPT resistance in *S. aureus* at least as far back as 1998 (predominantly in ST228). Therefore it is likely that these lineages express CPT resistance as a result of natural variation (and thus by chance) and/or were selected by environmental factors other than CPT. Since CPT itself has a low potential to select for resistance, Kelley et al. (2015) also speculated that other agents like β-lactam antibiotics may have contributed to the selection of this resistance phenotype (Knight et al., 2012; Mobegi et al., 2017). Supporting this hypothesis, we observed a positive correlation between the levels of CPT and OXA MICs (*p* < 0.001; *R*2 = 0.065; Supplementary Figure 6).

## Conclusion

With this study, we confirmed the potential of microbial GWAS to identify genetic variants that are significantly associated with antibiotic resistance in *S. aureus*. However, due to the clonal population structure of bacterial populations, it remains challenging to control for the detection of spurious associations. Therefore, profound sampling strategies are necessary to investigate the genetic architecture of bacterial phenotypes. The ongoing development and refinement of new and existing tools will enable researchers to account for stratification more precisely and to use multiple variants in association testing. A key challenge in microbial GWAS has been and still is the requirement for larger and more complex sample collections but is often restricted by the number of well-characterized clinical isolates available. A large collection not only increases the statistical power of association studies but also enables the investigation of traits that are linked to lineage-specific features and/or variants that occur with a low frequency.

## Data Availability Statement

The datasets generated for this study can be found in the online repositories. The names of the repository/repositories and accession number(s) can be found below: https://www.ebi.ac.uk/ena, PRJEB41643.

## Author Contributions

BS, GW, and SF: conception and design of the study. RW: strain characterization and manuscript writing. RW, JB, and AT: WGS sequencing. RW and AS: data curation and analyses. All authors: manuscript editing and reviewing.

## Conflict of Interest

The authors declare that the research was conducted in the absence of any commercial or financial relationships that could be construed as a potential conflict of interest.

## Footnotes

1 https://eucast.org/clinical_breakpoints/

2 https://www.cog-genomics.org/plink2

3 https://github.com/nvalimak/fsm-lite

4 http://mesquiteproject.org/

5 https://cge.cbs.dtu.dk/services/SCCmecFinder-1.0/

6 GitHub. SEER. https://github.com/johnlees/seer/wiki/Usage [Accessed November 30, 2020].

## Supplementary Material

Supplementary Figure 1 Distribution of resistance phenotypes for the DAP **(A)** and CPT **(B)** strain collection. Clonal lineages with corresponding numbers of included isolates are shown below. Resistant strains are highlighted as dark shaded columns, while susceptible isolates are shown as light shaded columns. The distribution of phenotypes is given in percent. DAP-R, daptomycin-resistant; DAP-S, daptomycin-susceptible; CPT-R, ceftaroline-resistant; CPT-S, ceftaroline-susceptible; ST, sequence type; CC, clonal cluster.

Supplementary Figure 2 Phylogenetic analysis of 95 *S. aureus* strains. Neighbor-Joining tree based on 7.342 SNPs describing the linear evolution among 95 *S. aureus* isolates. SNPs were called with an exclusion distance of 150 bp. Modified NC-002951 was used as a reference genome. hierBAPS clusters are represented by color-shaded boxes. Bold isolate identifiers indicate resistance towards DAP. Bootstraps are color-coded (minimum = red, maximum = green).

Supplementary Figure 3 Phylogenetic analysis of 87 *S. aureus* isolates. Neighbor-Joining tree based on 4.344 SNPs describing the linear evolution among 87 *S. aureus* isolates. SNPs were called with an exclusion distance of 150 bp. Modified NC-017343 was used as a reference genome. hierBAPS clusters are represented by color-shaded boxes. Bold isolate identifiers indicate resistance against CPT. Bootstraps are color-coded (minimum = red, maximum = green).

Supplementary Figure 4 Q-Q plot illustrating population stratification in GWAS. The plot compares the observed and expected *p*-value under the null hypothesis of no true association. Deviation from the X = Y reference line indicates the presence of a systematic bias. **(A)** DAP Q-Q plot with unadjusted PLINK *p*-values. **(B)** CPT Q-Q plot with GC-adjusted PLINK *p*-values. **(C)** DAP Q-Q plot with lrt corrected SEER *p*-values. **(D)** CPT Q-Q plot with Wald-corrected SEER *p*-values.

Supplementary Figure 5 Q-Q plot illustrating population stratification CPT GWAS. The plot compares the observed and expected *p*-value under the null hypothesis of no true association. Deviation from the X = Y reference line indicates the presence of a systematic bias. CPT Q-Q plot with unadjusted PLINK *p*-values **(A)** and lrt corrected SEER *p*-values **(B)**.

Supplementary Figure 6 Linear regression model fitted on the log_2_ values of CPT and OXA MICs of 98 *S. aureus* isolates (*p*-value < 0.001; R^2^ = 0.065). The horizontal and vertical dotted lines represent the OXA and CPT breakpoint for *S. aureus* respectively (according to EUCAST v. 7.1; OXA: S ≤ 2 mg/L, R > 2 mg/L; CPT: S ≤ 1 mg/L, R > 1 mg/L).

Supplementary Figure 7 Mauve genome alignment for the illustration of *mprF* sequence similarity. To illustrate the *mprF* sequence similarity between different clonal lineages, one representative DAP-R isolate was chosen from each *hier*BAPS subcluster. The *mprF* sequence was identified and extracted from *de novo* assembled contigs. To compute a genome alignment, we used Mauve and Geneious Prime v. 11.0.4. The following isolates were used: 14-02100 (ST45), 10-00785 (ST22), 13-04344 (ST225), 13-02671 (ST8), and 13-03385 (ST7). Nucleotide differences are highlighted as dark shaded boxes.

Supplementary Table 1 Details on the daptomycin strain collection.

Supplementary Table 2 Details on the ceftaroline strain collection.

Supplementary Table 3 Genetic elements excised from the reference sequences of *S. aureus* COL and *S. aureus* ECT-R2.

Supplementary Table 4 PLINK defined population outliers.

Supplementary Table 5 Detection of GrlA S80F and GyrA S84L.

Supplementary Table 6 Detection of *mecI*, *ΔmecRI*, and *mecRI* mutations.
