# Corrigendum: Genome-Wide Association Studies for the Detection of Genetic Variants Associated With Daptomycin and Ceftaroline Resistance in *Staphylococcus aureus*

**DOI:** 10.3389/fmicb.2021.686197

**Published:** 2021-04-27

**Authors:** Robert E. Weber, Stephan Fuchs, Franziska Layer, Anna Sommer, Jennifer K. Bender, Andrea Thürmer, Guido Werner, Birgit Strommenger

**Affiliations:** ^1^Department of Infectious Diseases, Robert Koch-Institute, Wernigerode, Germany; ^2^Methodology and Research Infrastructure, Bioinformatics, Robert Koch-Institute, Berlin, Germany; ^3^Methodology and Research Infrastructure, Genome Sequencing, Robert Koch-Institute, Berlin, Germany

**Keywords:** GWAS, daptomycin, ceftaroline, *S. aureus*, antibiotic resistance, PLINK, SEER

In the published article, there was an error in affiliations. Instead of “Robert E. Weber^1, 2^, Stephan Fuchs^3^, Franziska Layer^1, 2^, Anna Sommer^1, 2^, Jennifer K. Bender^1, 2^, Andrea Thürmer^3^, Guido Werner^1, 2^ and Birgit Strommenger^1, 2*^

^1^ Department of Infectious Diseases, Robert Koch-Institute, Wernigerode, Germany

^2^ Methodology and Research Infrastructure, Genome Sequencing, Robert Koch-Institute, Berlin, Germany

^3^ Methodology and Research Infrastructure, Bioinformatics, Robert Koch-Institute, Berlin, Germany”, it should be

“Robert E. Weber^1^, Stephan Fuchs^2^, Franziska Layer^1^, Anna Sommer^1^, Jennifer K. Bender^1^, Andrea Thürmer^3^, Guido Werner^1^ and Birgit Strommenger^1^

^1^ Department of Infectious Diseases, Robert Koch-Institute, Wernigerode, Germany

^2^ Methodology and Research Infrastructure, Bioinformatics, Robert Koch-Institute, Berlin, Germany

^3^ Methodology and Research Infrastructure, Genome Sequencing, Robert Koch-Institute, Berlin, Germany”

The authors apologize for this error and state that this does not change the scientific conclusions of the article in any way. The original article has been updated.

