# Supplementary figures and images for: Genome-Wide Association Studies for the Detection of Genetic Variants Associated With Daptomycin and Ceftaroline Resistance in Staphylococcus aureus
Source: Front Microbiol. 2021 Feb 15;12:639660. doi: 10.3389/fmicb.2021.639660 (PMC7917082; doi:10.3389/fmicb.2021.639660)

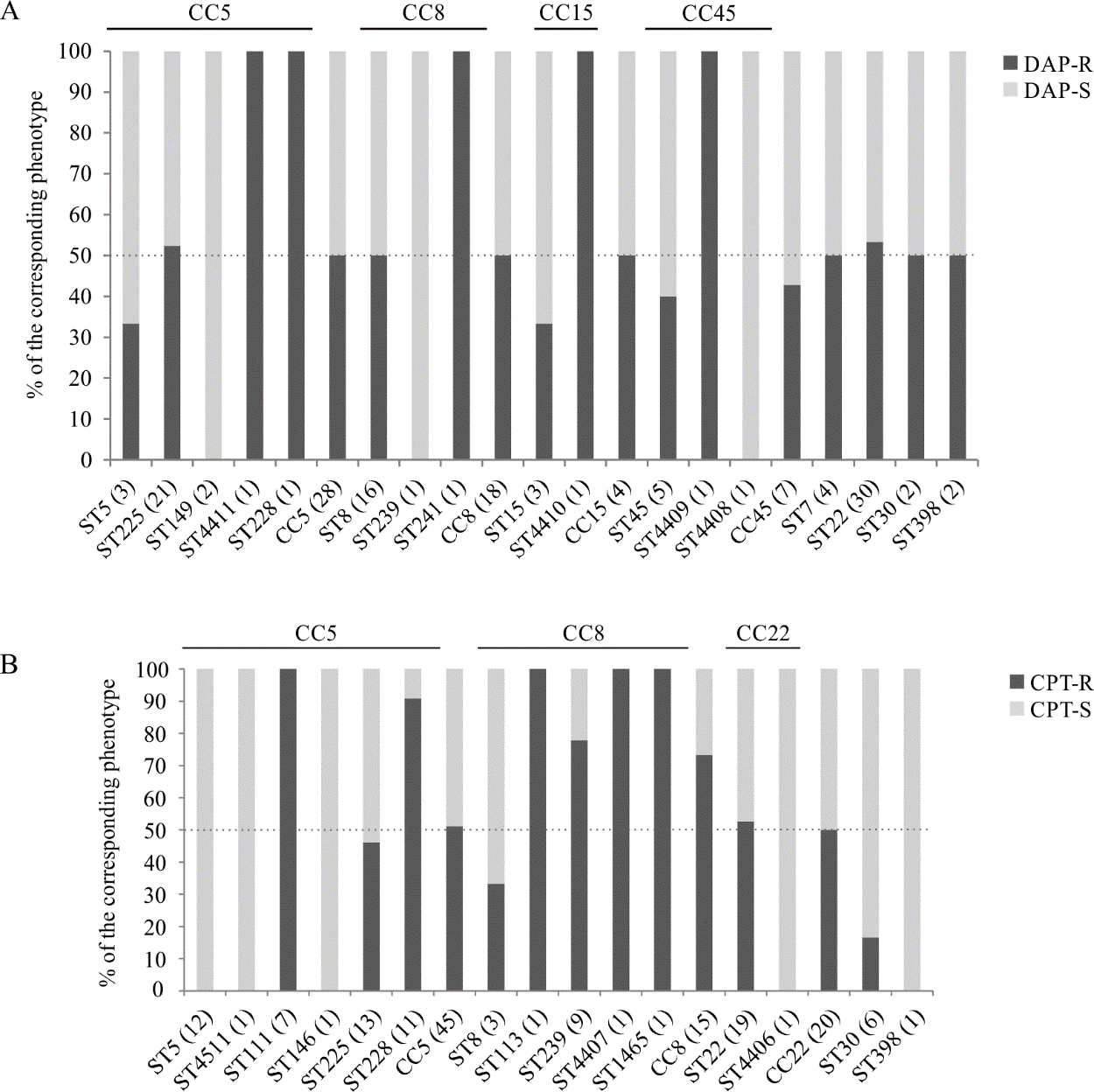

Supplement: Supplementary Figure 1 — Distribution of resistance phenotypes for the DAP (A) and CPT (B) strain collection. Clonal lineages with corresponding numbers of included isolates are shown below. Resistant strains are highlighted as dark shaded columns, while susceptible isolates are shown as light shaded columns. The distribution of phenotypes is given in percent. DAP-R, daptomycin-resistant; DAP-S, daptomycin-susceptible; CPT-R, ceftaroline-resistant; CPT-S, ceftaroline-susceptible; ST, sequence type; CC, clonal cluster. [file Image_1.png]

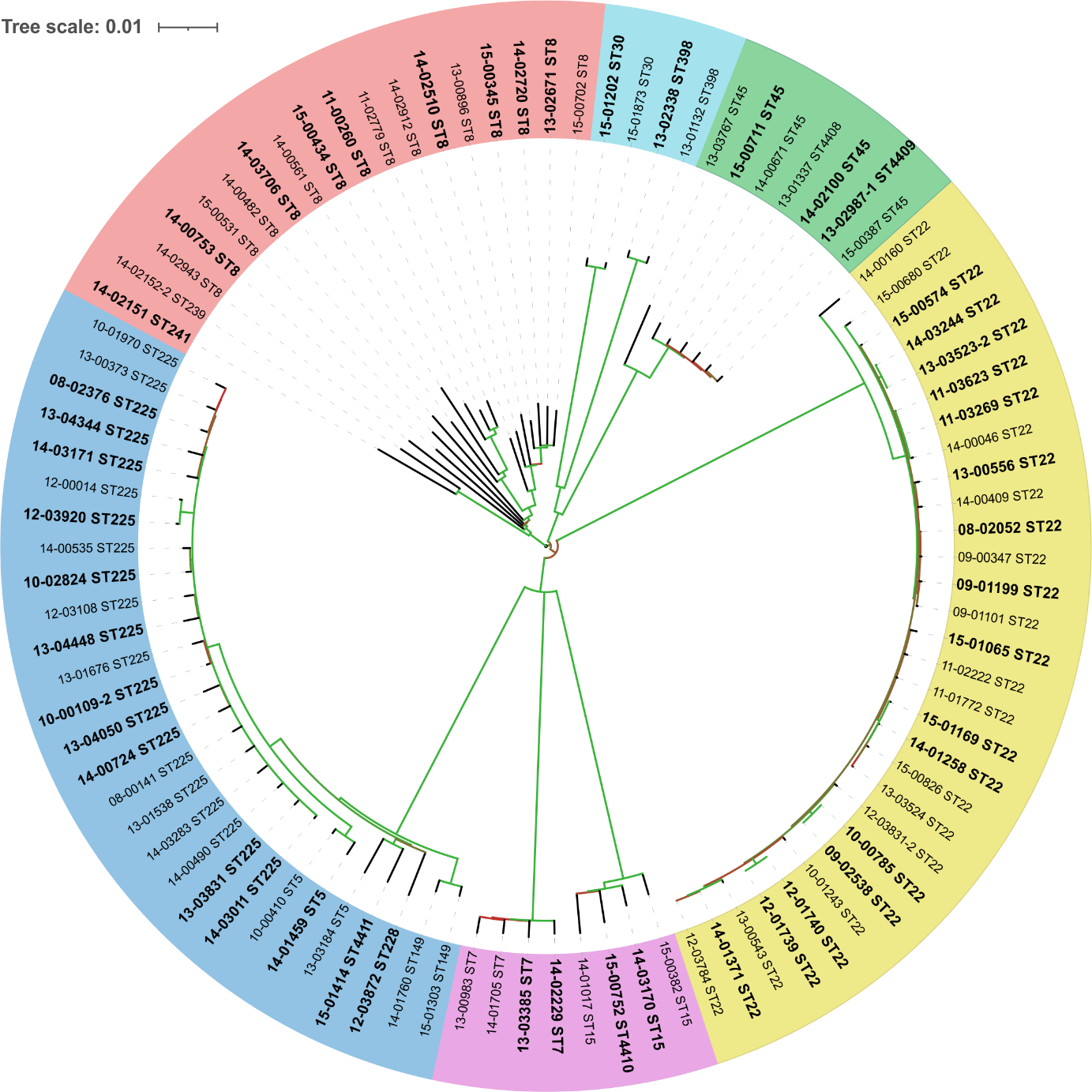

Supplement: Supplementary Figure 2 — Phylogenetic analysis of 95 S. aureus strains. Neighbor-Joining tree based on 7.342 SNPs describing the linear evolution among 95 S. aureus isolates. SNPs were called with an exclusion distance of 150 bp. Modified NC-002951 was used as a reference genome. hierBAPS clusters are represented by color-shaded boxes. Bold isolate identifiers indicate resistance towards DAP. Bootstraps are color-coded (minimum = red, maximum = green). [file Image_2.png]

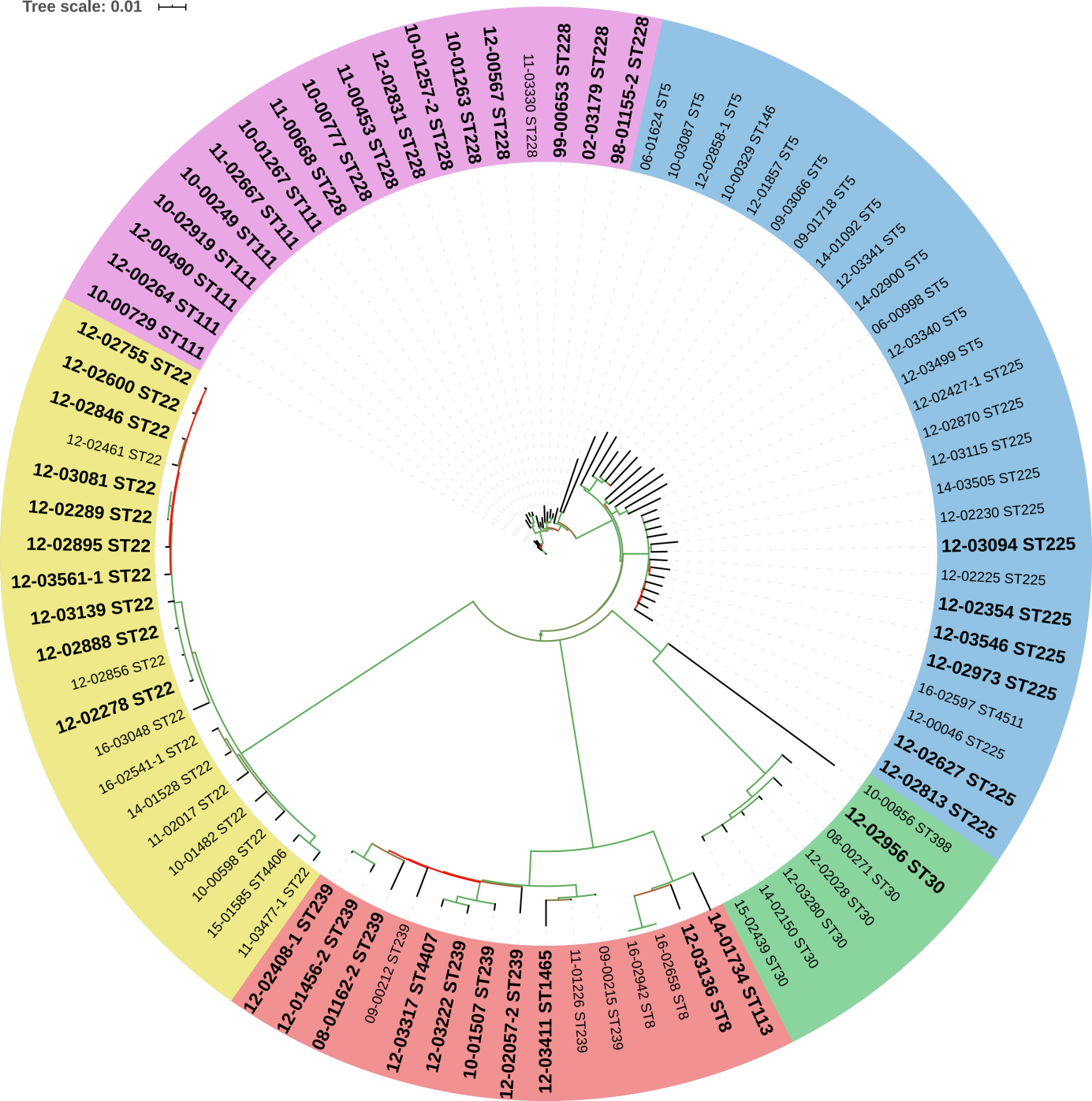

Supplement: Supplementary Figure 3 — Phylogenetic analysis of 87 S. aureus isolates. Neighbor-Joining tree based on 4.344 SNPs describing the linear evolution among 87 S. aureus isolates. SNPs were called with an exclusion distance of 150 bp. Modified NC-017343 was used as a reference genome. hierBAPS clusters are represented by color-shaded boxes. Bold isolate identifiers indicate resistance against CPT. Bootstraps are color-coded (minimum = red, maximum = green). [file Image_3.png]

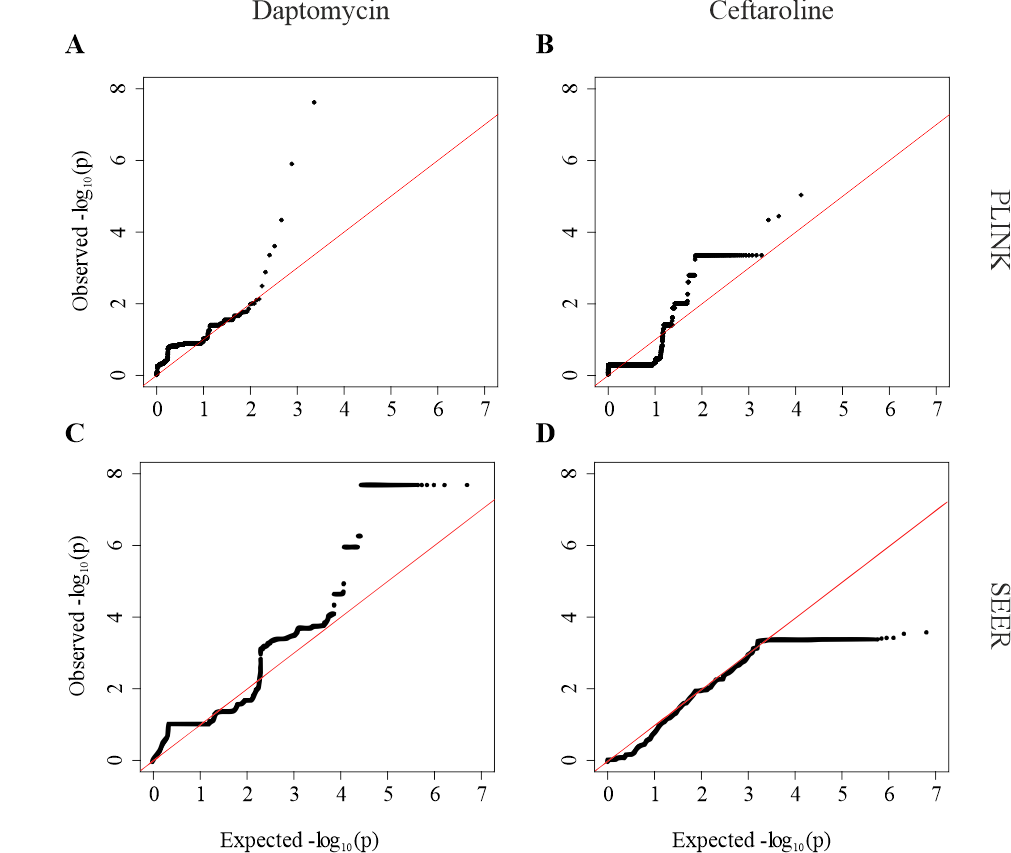

Supplement: Supplementary Figure 4 — Q-Q plot illustrating population stratification in GWAS. The plot compares the observed and expected p-value under the null hypothesis of no true association. Deviation from the X = Y reference line indicates the presence of a systematic bias. (A) DAP Q-Q plot with unadjusted PLINK p-values. (B) CPT Q-Q plot with GC-adjusted PLINK p-values. (C) DAP Q-Q plot with lrt corrected SEER p-values. (D) CPT Q-Q plot with Wald-corrected SEER p-values. [file Image_4.png]

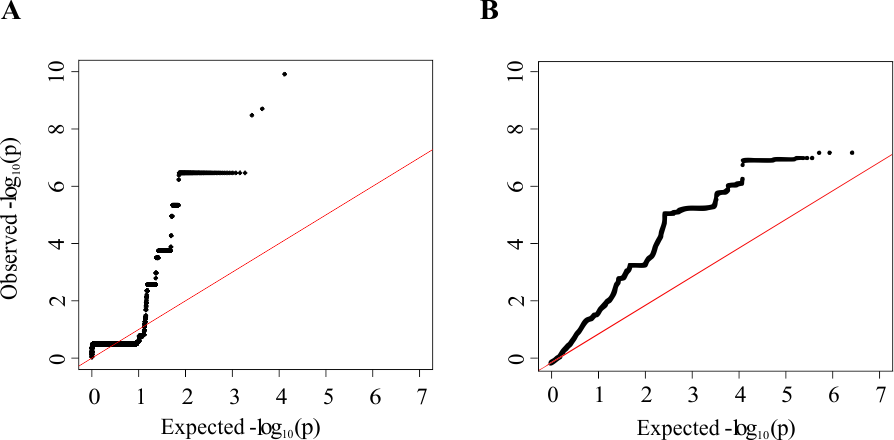

Supplement: Supplementary Figure 5 — Q-Q plot illustrating population stratification CPT GWAS. The plot compares the observed and expected p-value under the null hypothesis of no true association. Deviation from the X = Y reference line indicates the presence of a systematic bias. CPT Q-Q plot with unadjusted PLINK p-values (A) and lrt corrected SEER p-values (B). [file Image_5.png]

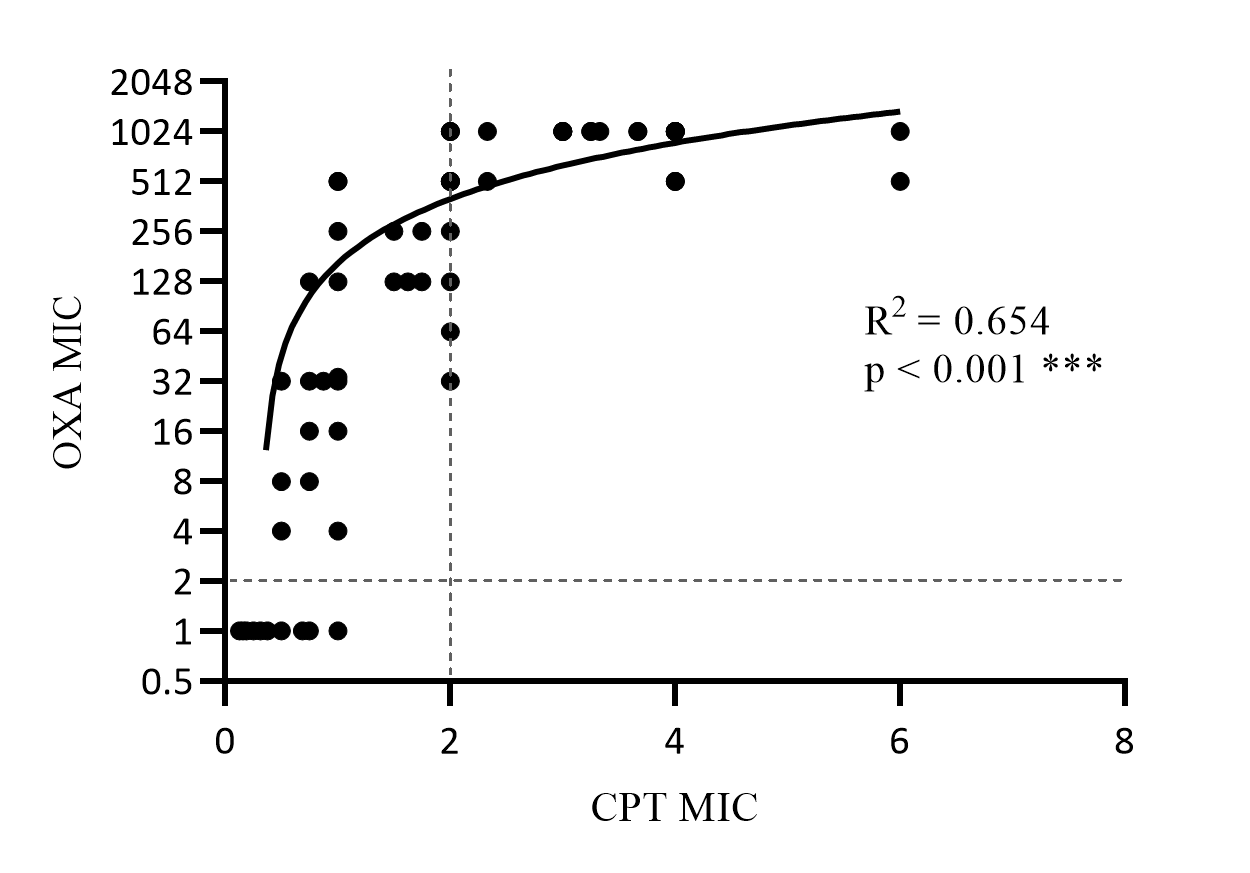

Supplement: Supplementary Figure 6 — Linear regression model fitted on the log2 values of CPT and OXA MICs of 98 S. aureus isolates (p-value < 0.001; R2 = 0.065). The horizontal and vertical dotted lines represent the OXA and CPT breakpoint for S. aureus respectively (according to EUCAST v. 7.1; OXA: S ≤ 2 mg/L, R > 2 mg/L; CPT: S ≤ 1 mg/L, R > 1 mg/L). [file Image_6.png]

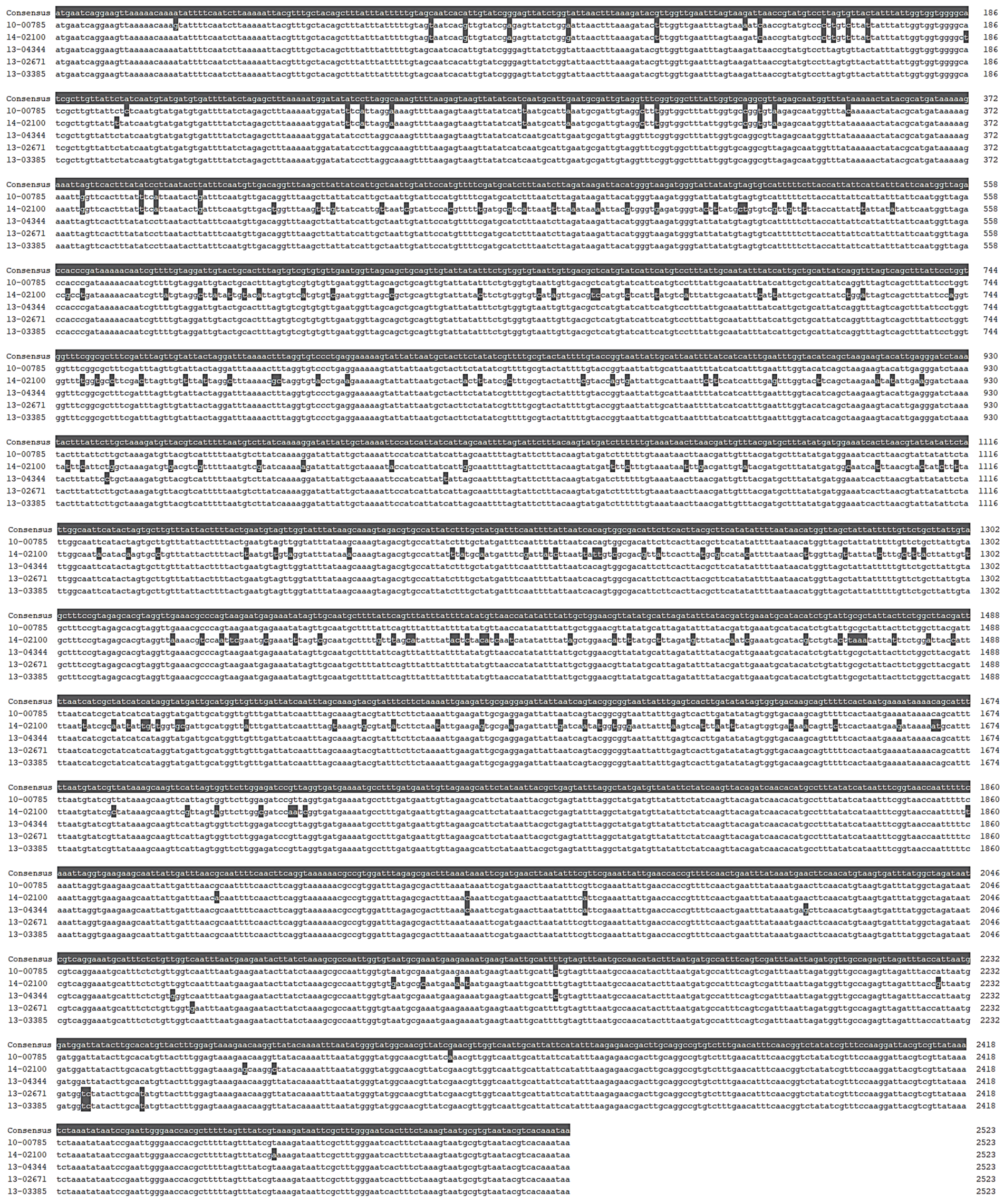

Supplement: Supplementary Figure 7 — Mauve genome alignment for the illustration of mprF sequence similarity. To illustrate the mprF sequence similarity between different clonal lineages, one representative DAP-R isolate was chosen from each hierBAPS subcluster. The mprF sequence was identified and extracted from de novo assembled contigs. To compute a genome alignment, we used Mauve and Geneious Prime v. 11.0.4. The following isolates were used: 14-02100 (ST45), 10-00785 (ST22), 13-04344 (ST225), 13-02671 (ST8), and 13-03385 (ST7). Nucleotide differences are highlighted as dark shaded boxes. [file Image_7.png]
